# Health resource utilization associated with skeletal-related events: results from a retrospective European study

**DOI:** 10.1007/s10198-015-0716-7

**Published:** 2015-08-08

**Authors:** Jean-Jacques Body, João Pereira, Harm Sleeboom, Nikos Maniadakis, Evangelos Terpos, Yves Pascal Acklin, Jindrich Finek, Oliver Gunther, Guy Hechmati, Tony Mossman, Luis Costa, Wojciech Rogowski, Hareth Nahi, Roger von Moos

**Affiliations:** CHU Brugmann, Université Libre de Bruxelles, Brussels, Belgium; Universidade Nova de Lisboa, National School of Public Health, Lisbon, Portugal; Haga Hospital, The Hague, The Netherlands; Department of Health Services Management, National School of Public Health, Athens, Greece; University of Athens School of Medicine, Alexandra University Hospital, Athens, Greece; Kantonsspital Graubünden, Chur, Switzerland; University Hospital, Pilsen, Czech Republic; Centre for Observational Research, Amgen Ltd, Uxbridge, UK; Health Economics, Amgen (Europe) GmbH, Zug, Switzerland; Biostatistics, Amgen Ltd, Cambridge, UK; Serviço de Oncologia do Hospital de Santa Maria, Instituto de Medicina Molecular, Lisbon, Portugal; ZOZ MSWiA Z Warmińsko Mazurskim Centrum Onkologii, Olszytn, Poland; Division of Hematology, Department of Medicine, Karolinska Institute, Huddinge, Stockholm, Sweden

**Keywords:** Health resource utilization (HRU), Skeletal-related event (SRE), Bone metastases, Breast cancer, Prostate cancer, Lung cancer

## Abstract

**Background:**

Bone complications, also known as skeletal-related events (SREs), are common in patients with bone metastases secondary to advanced cancers.

**Objective:**

To provide a detailed estimate of the health resource utilization (HRU) burden associated with SREs across eight European countries.

**Methods:**

Eligible patients from centers in Austria, the Czech Republic, Finland, Greece, Poland, Portugal, Sweden, and Switzerland with bone metastases or lesions secondary to breast cancer, prostate, or lung cancer or multiple myeloma who had experienced at least one SRE (defined as radiation to bone, long-bone pathologic fracture, other bone pathologic fracture, surgery to bone or spinal cord compression) were entered into this study. HRU data were extracted retrospectively from the patients’ charts from 3.5 months before the index SRE until 3 months after the index SRE (defined as an SRE preceded by an SRE-free period of at least 6.5 months).

**Results:**

Overall, the mean number of inpatient stays per SRE increased from baseline by approximately 0.5–1.5 stays, with increases in the total duration of inpatient stays of approximately 6–37 days per event. All SREs were associated with substantial increases from baseline in the frequency of procedures and the number of outpatient and day-care visits.

**Conclusions:**

SREs are associated with substantial HRU owing to considerable increases in the number and duration of inpatient stays, and in the number of procedures, outpatient visits, and day-care visits. These data collectively provide a valuable summary of the real-world SRE burden on European healthcare systems.

## Introduction

The progression of cancer to the skeleton is a common occurrence in patients with advanced disease; at post-mortem examination, 65–90 % of patients with breast or prostate cancer and approximately 35 % of individuals with lung cancer have bone metastases [1, 2] and almost all patients with multiple myeloma develop bone lesions [3]. Metastatic bone disease is the cause of considerable morbidity [2], with affected patients at high risk of experiencing bone complications, also referred to as skeletal-related events (SREs), including radiation to bone, pathologic fracture, surgery to bone, and spinal cord compression [4]. Unless patients are treated with a bone-targeting agent (BTA), SREs may occur as frequently as every 3–6 months [2]. As mobility and functional independence diminish with subsequent SREs, overall health-related quality of life also declines [4]. Furthermore, patients with metastatic bone disease and an SRE have a poorer prognosis and increased risk of death compared with patients who are SRE naïve [5–7].

Following an SRE, patient care and treatment can be costly, as well as placing a considerable and complex demand on healthcare resources [8–10]. A retrospective analysis from the Netherlands estimated that the mean per patient cost to treat SREs in individuals with prostate cancer and bone metastases was €6973 (range, €1187–€40,948) [11]. Despite the differences in the healthcare systems in the Netherlands and the UK, similar values have been reported for patients in the UK with breast cancer and bone metastases, with an estimated mean lifetime SRE-associated cost of £11,314–£19,121 (€14,029–€23,710; 1 GBP = 1.24 EUR) [12]. Notably, total medical care costs are substantially higher for patients who have bone metastases and one or more SREs than for those with bone metastases and no SREs (estimated US$48,173 [€37,093; 1 US$ = 0.77 EUR] more per patient per 60 months in the USA) [13].

Although some studies have attempted to estimate SRE-associated costs, there are a limited number of analyses that review specific health resource utilization (HRU) associated with SREs, particularly at a country level within Europe. An analysis from Spain found that patients (*N* = 28,162) with bone metastases and an SRE required a greater duration of hospital stays and a greater duration of hospital stays due to re-admission, than did patients with bone metastases without SREs [10]. Similarly, patients with bone metastases and SREs had a greater number of hospital readmissions than patients with a primary cancer diagnosis but no metastatic bone disease, suggesting that as the disease progresses, there is a greater HRU burden [10]. In Portugal, a limited retrospective chart review of patients with breast cancer (*n* = 121) or prostate cancer (*n* = 31) and at least one SRE occurring within 12 months reported high costs associated with SREs; these costs were predominantly due to hospitalization and medication [8]. In the USA, a prospective study of 238 patients reported substantial HRU associated with SREs, with considerable numbers and durations of inpatient stays, numbers of outpatient visits, and numbers of procedures [9]. The same was concluded from the European cohort of the same study (conducted in Germany, Italy, Spain, and the UK; *n* = 631) in which all SREs were associated with considerable HRU burden and costs [14, 15].

The availability of country-specific HRU data would help to describe the burden of SREs on individual European healthcare systems and might help when assessing the overall value of new treatment options. These data are of particular relevance given the resource constraints under which many healthcare systems now operate, as they would allow an accurate estimation of resources required to manage patients with SREs and could be used to determine SRE-associated costs for use in budgeting. Thus, this study was conducted to provide country-specific estimates of HRU associated with SREs in eight European countries, where robust data were previously unavailable.

## Methods

### Study design

This was a multinational, before-and-after, retrospective study that enrolled patients from hospitals in Austria, the Czech Republic, Finland, Greece, Poland, Portugal, Sweden, and Switzerland.

### Patients

Eligible patients were aged 20 years or older, had bone metastases secondary to breast, lung or prostate cancer or bone lesions due to multiple myeloma, and had at least one index SRE (an SRE preceded by an SRE-free period of at least 6.5 months) within the 5-year time period between July 1, 2004 and July 1, 2009. Patients were excluded from the study if they were participating or had previously participated in a denosumab clinical trial, died less than 2 weeks after the index SRE, or had chart data that were of insufficient quality.

### SRE data collection

**Fig. 1:**
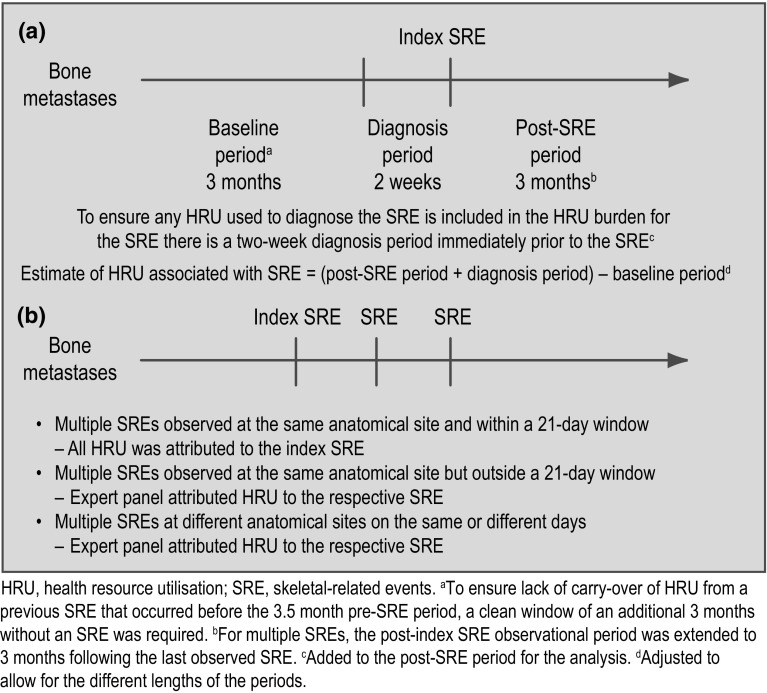
Study design and data collection for patients with one SRE (**a**), and multiple SREs (**b**)

SREs were defined as radiation to bone, pathologic fracture (of long or other bone), surgery to bone or spinal cord compression. To ensure a representative distribution of SREs in each country, a target maximum number of each type of index SRE was assigned. Per country, this target was 150 patients to reach the required 150 index SREs (one index SRE per patient) as follows: radiation therapy to bone (*n* = 60); long-bone pathologic fracture (*n* = 30); other bone pathologic fracture (*n* = 30); surgery to bone (*n* = 20); and spinal cord compression (*n* = 10). For all SREs, data were extracted from patients’ hospital charts beginning 3.5 months before the index SRE until 3 months after the index SRE (Fig. 1a). Recruitment ceased when the pre-specified target was reached for each type of index SRE. Once a patient was enrolled into the study, data were captured for all SREs occurring during the period after the index SRE. For patients who experienced multiple SREs, the data extraction period was extended to 3 months after the last SRE that the patient experienced during the study period (Fig. 1b). There was no limit to the number of SREs included in the period after the index SRE, as long as the SRE occurred within the 5-year inclusion period. In addition, patients’ baseline clinical and demographic characteristics were captured.

### Data collection and attribution of HRU

For single SREs, HRU was objectively attributed according to study design: a period of 3 months, starting 3.5 months before the index SRE, was used to establish baseline HRU and the 14 day (0.5 month) period immediately before the index SRE was used to estimate diagnostic HRU. To ensure that there was no carry-over of HRU from any SREs that occurred before the 3.5 month pre-index-SRE period, a preceding SRE-free period of a further 3 months was required (Fig. 1a). Adjustments were made to account for differences in the lengths of baseline and post-baseline periods. If multiple SREs were present at the same anatomical site and within 21 days of the index SRE, all HRU was attributed to the index SRE (Fig. 1b). If multiple SREs were observed at the same anatomical site but outside the 21-day window, or at different anatomical sites on the same or different days, the study steering committee, comprising four clinicians and two health economists (authors of this paper), attributed HRU to the appropriate SRE based on their experience and opinion. The steering committee was required to attribute HRU to an SRE in only approximately 5 % of cases.

Primary HRU outcome measures recorded were: number and duration of inpatient hospital stays (overall and by hospital unit type); number of procedures (overall and by provider type); number of emergency room visits; number of outpatient visits; and number of day-care hospital visits. Outpatient visits and procedures were reviewed by an expert panel to separate SRE-associated HRU from HRU related to the management of the underlying cancer.

### Statistical analysis

Statistical analyses were descriptive in nature and summarized numbers of patients along with mean, median, standard deviation (SD), quartile, minimum and maximum values. Patient characteristics and HRU outcome measures were summarized overall and by index SRE and subsequent SRE type. Data are presented as mean (SD), unless stated otherwise, because this describes the total resources used at a population level better than the other values, and resource use at this level is key to making healthcare policy decisions.

After adjustment for differences in the lengths of the baseline and post-baseline periods, the change from baseline was used to estimate HRU associated with each SRE.

## Results

### Study population

A total of 1022 patients were included from the eight countries. Baseline demographics and clinical characteristics are presented for the overall population and by country (Table 1).

**Table 1:** Baseline demographics and clinical characteristics

	All countries (*N* = 1022)	Austria (*n* = 131)	Czech Republic (*n* = 130)	Finland (*n* = 117)	Greece (*n* = 121)	Poland (*n* = 150)	Portugal (*n* = 126)	Sweden (*n* = 119)	Switzerland (*n* = 128)
Mean age, years	63.8	61.2	64.3	66.4	61.8	61.9	63.2	67.9	64.3
Female, *n* (%)	467 (45.7)	82 (62.6)	71 (54.6)	43 (36.8)	47 (38.8)	80 (53.3)	55 (43.7)	29 (24.4)	60 (64.9)
Geriatric age group, *n* (%)									
<65 years	522 (51.1)	79 (60.3)	61 (46.9)	49 (41.9)	64 (52.9)	90 (60.0)	67 (53.2)	46 (38.7)	66 (51.6)
≥65 years	500 (48.9)	52 (39.7)	69 (53.1)	68 (58.1)	57 (47.1)	60 (40.0)	59 (46.8)	73 (61.3)	62 (48.4)
≥75 years	179 (17.5)	17 (13.0)	20 (15.4)	22 (18.8)	13 (10.7)	20 (13.3)	22 (17.5)	40 (33.6)	25 (19.5)
Radiation therapy, *n* (%)	482 (47.2)	57 (43.5)	59 (45.4)	60 (51.3)	59 (48.8)	67 (44.7)	59 (46.8)	62 (52.1)	59 (46.1)
Pathologic fracture (long bone),* n* (%)	118 (11.5)	25 (19.1)	18 (13.8)	8 (6.8)	8 (6.6)	28 (18.7)	14 (11.1)	9 (7.6)	8 (6.3)
Pathologic fracture (other bone), *n* (%)	241 (23.6)	22 (16.8)	33 (25.4)	30 (25.6)	32 (26.4)	30 (20.0)	29 (23.0)	27 (22.7)	38 (29.7)
Surgery to bone, *n* (%)	99 (9.7)	17 (13.0)	10 (7.7)	9 (7.7)	12 (9.9)	16 (10.7)	12 (9.5)	11 (9.2)	12 (9.4)
Spinal cord compression, *n* (%)	82 (8.0)	10 (7.6)	10 (7.7)	10 (8.5)	10 (8.3)	9 (6.0)	12 (9.5)	10 (8.4)	11 (8.6)
ECOG status^a^ *n* (%)									
0	107 (13.8)	31 (43.1)	10 (8.7)	4 (4.3)	14 (13.6)	6 (4.1)	4 (5.8)	8 (13.1)	30 (26.3)
1	318 (41.1)	31 (43.1)	58 (50.4)	35 (37.2)	38 (36.9)	56 (38.4)	32 (46.4)	18 (29.5)	50 (43.9)
2	234 (30.2)	7 (9.7)	34 (29.6)	36 (38.2)	41 (39.8)	56 (38.4)	16 (23.2)	20 (32.8)	24 (21.1)
3	103 (13.3)	3 (4.2)	10 (8.7)	19 (20.2)	9 (8.7)	25 (17.1)	14 (20.3)	13 (21.3)	10 (8.8)
4	12 (1.6)	0 (0.0)	3 (2.6)	0 (0.0)	1 (1.0)	3 (2.1)	3 (4.3)	2 (3.3)	0 (0.0)
Unknown	248 (–)	59 (–)	15 (–)	23 (–)	18 (–)	4 (–)	57 (–)	58 (–)	14 (–)
Primary tumor diagnosis, *n* (%)									
Breast cancer	321 (31.4)	69 (52.7)	69 (53.1)	26 (22.2)	19 (15.7)	50 (33.3)	41 (32.5)	6 (5.0)	41 (32.0)
Lung cancer	184 (18.0)	25 (19.1)	7 (5.4)	19 (16.2)	41 (33.9)	31 (20.7)	41 (32.5)	4 (3.4)	16 (12.5)
Prostate cancer	267 (26.1)	6 (4.6)	46 (35.4)	49 (41.9)	12 (9.9)	28 (18.7)	31 (24.6)	59 (49.6)	36 (28.1)
Multiple myeloma	250 (24.5)	31 (23.7)	8 (6.2)	23 (19.7)	49 (40.5)	41 (27.3)	13 (10.3)	50 (42.0)	35 (27.3)
SRE status, *n* (%)									
Single	597 (58.4)	73 (55.7)	93 (71.5)	41 (35.0)	85 (70.2)	97 (64.7)	70 (55.6)	60 (50.4)	78 (60.9)
Multiple	425 (41.6)	58 (44.3)	37 (28.5)	76 (65.0)	36 (29.8)	53 (35.3)	56 (44.4)	59 (49.6)	50 (39.1)
Time since bone metastases, months									
*n*	754	88	120	94	71	109	111	69	92
Mean	11.14	8.30	13.48	16.72	6.01	4.67	6.24	16.37	18.76
Median	1.82	1.45	3.79	3.14	1.12	1.02	1.18	7.98	6.74
Bone metastases sites^a^, *n* (%)									
1–2	634 (82.1)	91 (91.0)	91 (74.6)	57 (60.6)	67 (93.1)	102 (93.6)	113 (100)	48 (69.6)	65 (69.9)
3–4	66 (8.5)	6 (6.0)	19 (15.6)	15 (16.0)	3 (4.2)	3 (2.8)	0 (0.0)	12 (17.4)	8 (8.6)
≥5	72 (9.3)	3 (3.0)	12 (9.8)	22 (23.4)	2 (2.8)	4 (3.7)	0 (0.0)	9 (13.0)	20 (21.5)
Missing	250 (–)	31 (–)	8 (–)	23 (–)	49 (–)	41 (–)	13 (–)	50 (–)	35 (–)
Bisphosphonate use, *n* (%)									
Baseline	330 (40.2)	36 (27.5)	62 (47.7)	47 (40.2)	23 (19.0)	49 (32.7)	30 (23.8)	35 (29.4)	48 (37.5)
Post-SRE	644 (63.0)	84 (64.1)	101 (77.7)	80 (68.4)	64 (52.9)	95 (63.3)	76 (60.3)	53 (44.5)	91 (71.1)

*ECOG* Eastern Cooperative Oncology Group

^a^Percentages calculated for the number of subjects with available data

The proportion of female patients was notably lower in Sweden (24.4 %) and higher in Switzerland (64.9 %) and Austria (62.6 %) compared with the overall proportion across all countries (45.7 %). Similarly, differences were seen in the proportions of patients with each primary tumor type across the countries: fewer enrolled patients had breast cancer in Sweden (5.0 %) than in Austria (52.7 %) or the Czech Republic (53.1 %); fewer enrolled patients in Sweden (3.4 %) and the Czech Republic (5.4 %) had lung cancer than in the other countries; and a smaller proportion of patients in Austria (4.6 %) and Greece (9.9 %) had prostate cancer than in Sweden (49.6 %).

In general, the majority of SREs recorded were single events, with the exception of Finland where single SREs accounted for 35.0 % of SREs. There was some variation between countries in the mean time since diagnosis of bone metastases, ranging from 4.7 months in Poland to 18.8 month in Switzerland).

### Changes in the number and duration of inpatient stays

**Fig. 2:**
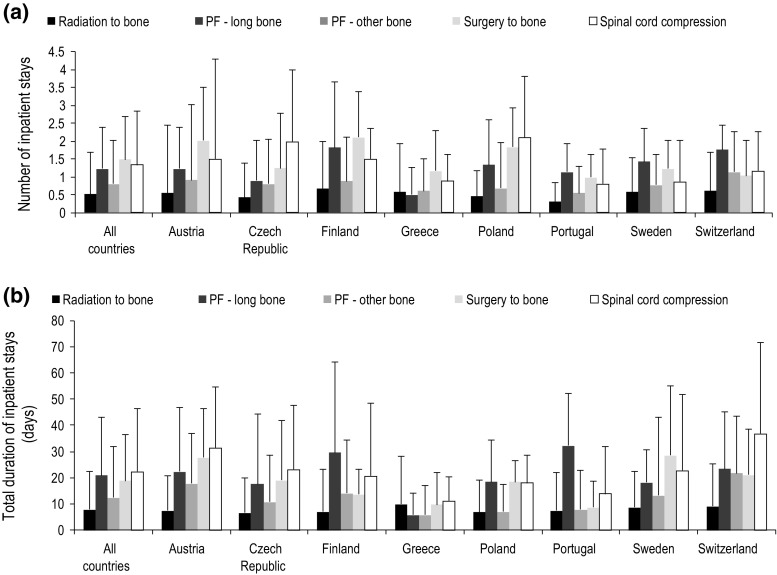
Mean (SD) change from baseline for number (**a**), and total duration (**b**) inpatient stays. *PF* pathologic fracture

Overall, an additional mean 0.5 (SD 1.2) inpatient stay was required per radiation to bone event compared with baseline. The corresponding increases for other SRE events were: 1.2 (1.2) for long-bone pathologic fractures; 0.8 (1.2) for other bone pathologic fractures; 1.5 (1.2) for surgery to bone events; and 1.3 (1.5) for spinal cord compressions (Fig. 2a).

The total duration of inpatient stays per SRE also increased compared with baseline, by: 7.8 (14.8) days for radiation to bone events; 20.9 (22.1) days for long-bone pathologic fractures, 12.3 (19.5) days for other bone pathologic fractures; 18.8 (17.5) days for surgery to bone events; and 22.2 (24.3) days for spinal cord compressions (Fig. 2b).

### Country-specific changes in the number and duration of inpatient stays

Changes from baseline in the number (Fig. 2a) and duration (Fig. 2b) of inpatient stays were generally similar across countries, with a requirement for an additional 0.5–2 stays per SRE (Fig. 2a). In Finland, Sweden, and Switzerland, long-bone pathologic fractures were associated with the greatest increase in number of inpatient stays, with mean (SD) increases of 1.8 (1.8), 1.4 (0.9), and 1.8 (0.7) stays per event, respectively. In Austria, Finland, and Greece, surgery to bone events were associated with the greatest increase in number of inpatient stays per event [mean (SD) increases of 2.0 (1.5), 2.1 (1.3) and 1.2 (1.1) stays, respectively]. For the Czech Republic and Poland, the SRE associated with the greatest increase in number of inpatient stays was spinal cord compression, with mean (SD) increases of 2.0 (2.0) and 2.1 (1.7) stays per event, respectively. In general, SREs treated in Greece and Portugal required fewer additional inpatient stays compared with the other countries studied.

Greater variation was observed in the increase from baseline in duration of inpatient stays compared with that seen in the number of inpatient stays (Fig. 2), ranging from approximately 6 to 37 days per SRE (Fig. 2b). The increase in duration of inpatient stays per event was generally smaller for radiation to bone events than for other SREs, and was similar across countries. In Finland, Poland and Portugal, the greatest increase in duration of inpatient stays per SRE was for long-bone pathologic fractures: mean (SD) increases of 29.4 (34.6), 18.5 (16.1), and 32.1 (19.8) days, respectively. In Sweden, surgery to bone resulted in the greatest mean (SD) increase in duration of inpatient stays, at 28.5 (26.5) additional days per event. In Austria, the Czech Republic, Greece and Switzerland, the increase in duration of inpatient stays was greatest for spinal cord compression, with mean (SD) increases of 31.4 (23.4), 23.1 (24.8), 11.1 (9.3), and 36.8 (35.0) days per event, respectively.

### Changes in the number of inpatient stays by hospital unit type

Increases from baseline in the number of inpatient stays per SRE most commonly involved [mean (SD) increase in number of stays]: oncology units [0.2 (0.7)] and radiation units [0.1 (0.4)] for radiation to bone events; orthopedic units [0.5 (0.6)] for long-bone pathologic fractures; internal medicine units [0.2 (0.6)] and oncology units [0.2 (0.7)] for other bone pathologic fractures; orthopedic units [0.6 (0.6)] for surgery to bone events and oncology units [0.3 (0.9)] for spinal cord compression.

Country-specific treatment practices were evident: in Austria, Finland and Greece, SREs were predominantly managed in orthopedic and oncology units, whereas in Sweden and Portugal a variety of specialist units were used, including urology and pneumology. In contrast to the other countries studied, in Switzerland the majority of SREs were managed in internal medicine units.

### Overall change in the number of procedures

**Fig. 3:**
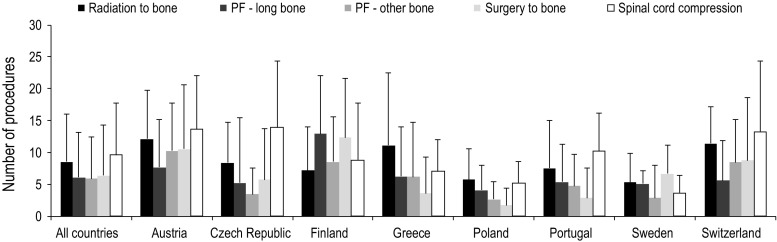
Mean (SD) change from baseline for number of procedures. *PF* pathologic fracture

Overall, all SREs required an increased number of procedures from baseline (Fig. 3). For radiation to bone events an additional mean (SD) of 8.5 (7.5) procedures were required per event. Long-bone pathologic fractures required an additional mean 6.1 (SD 7.1) procedures per event; this was similar for other bone pathologic fractures at 5.9 (6.6) additional procedures per event. Surgery to bone events required an additional mean 6.4 (SD 7.9) procedures and spinal cord compressions required 9.6 (8.2) additional procedures per event (Fig. 3). Increases in the frequency of procedures from baseline were generally of similar magnitude across all countries, ranging from approximately 2 to 14 procedures per SRE, although the increase was lower in Poland and Sweden than in the other countries (Fig. 3).

### Changes in emergency room visits

Overall, increases from baseline in the frequency of emergency room visits were relatively small, with an additional mean (SD) of 0.1 (0.7) visits per SRE for radiation to bone, 0.3 (0.7) for long-bone pathologic fractures, 0.2 (0.9) for other bone pathologic fractures, 0.2 (0.8) for surgery to bone and 0.5 (0.8) for spinal cord compressions. The greatest increases number of in emergency room visits per SRE were noted in Finland and Portugal, particularly associated with spinal cord compressions: mean (SD) of 1.2 (0.8) visits (Finland) and 1.1 (1.3) visits (Portugal).

### Changes in outpatient and day care visits

**Fig. 4:**
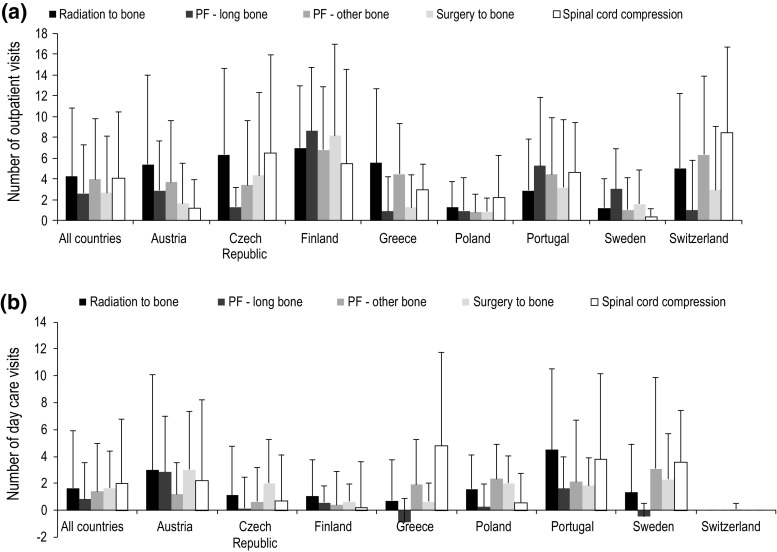
Mean (SD) change from baseline for number of outpatient visits (**a**) and number of day care visits (**b**). *PF* pathologic fracture

Collectively, outpatient visits increased in frequency from baseline by a mean (SD) of 4.2 (6.6) visits per SRE for radiation to bone events, 2.6 (4.7) for long-bone pathologic fractures, 4.0 (5.8) for other bone pathologic fractures, 2.7 (5.5) for surgery to bone events and 4.1 (6.4) for spinal cord compressions (Fig. 4a). Across countries, the increase from baseline in outpatient visits ranged from approximately 1 to 8 visits per SRE and was generally highest in Finland.

Day care visits also increased in overall frequency from baseline by a mean (SD) per SRE of 1.6 (4.3) for radiation to bone, 0.8 (2.7) for long-bone pathologic fractures, 1.4 (3.6) for other bone pathologic fractures, 1.7 (2.8) for surgery to bone events, and 2.0 (4.8) for spinal cord compressions (Fig. 4b). Across countries, day-care visits increased most notably in Portugal and Austria, with substantial increases also recorded in Sweden and Greece, particularly for spinal cord compression. Values of zero were recorded for Switzerland as these data were not available to investigators.

## Discussion

This study is the first multinational, European, before-and-after, retrospective study to describe SRE-associated HRU in real-world practice across a number of tumor types. All SREs were associated with substantial HRU, demonstrated by increases from baseline in the number and duration of inpatient stays, as well as in the number of procedures and outpatient, emergency room, and day-care visits. The primary strength of this study is that the HRU data captured here are representative of clinical practice across eight European countries.

In all countries, all SRE types were associated with increases from baseline in the number and duration of inpatient stays; however, these increases differed according to SRE type. For example, surgery to bone and spinal cord compression contributed up to three times more HRU than radiation to bone. Increases in inpatient HRU for pathologic fractures of long bones were similar to those for surgery to bone or spinal cord compression, and were greater than increases in HRU for fractures affecting other bones, perhaps because longer periods of immobility or more extensive medical interventions were required than for more minor fractures. Our data are consistent with those from other European studies that report substantial inpatient HRU as a result of surgery to bone, spinal cord compression and pathologic fracture [10, 15]; however, our study is the first to distinguish between pathologic fractures of long bones and those of other bones in terms of HRU in clinical practice.

Some variations in the pattern of HRU were observed in different countries, one example being the duration of inpatient stays: Greece and Poland generally had the smallest increases from baseline in duration of inpatient stays, and Switzerland had the largest increases. While small increases in the duration of inpatient stays across most SRE types were seen in Portugal, long-bone pathologic fracture was associated with one of the largest increases in mean duration of inpatient stays of all the countries. This may indicate a difference in the approach to care for this fracture type compared with other SREs in Portugal.

The majority of inter-country differences in HRU were observed in the change from baseline in the numbers of outpatient visits, day-care visits and procedures. For example, in Finland and Portugal, long-bone pathologic fracture accounted for the largest increase from baseline in outpatient HRU of all SREs, whereas in most other countries, this SRE type was associated with small increases in outpatient HRU. This suggests that there are differences in treatment practice, such as the use of less invasive surgical procedures that may account for the increased use of outpatient or day-care facilities in some countries. The increase in the use of outpatient or day-care clinics may also reflect the accessibility of such facilities in certain countries. If patients are required to travel long distances for treatment, this may make overnight stays necessary, thus increasing inpatient stays and concurrently decreasing outpatient visits. In Switzerland, Austria, and the Czech Republic, the largest increases in outpatient visits and number of procedures were for spinal cord compression, but in Greece, radiation to bone accounted for the largest changes. The increases in number of procedures and outpatient and day-care visits associated with radiation to bone in many countries is perhaps unsurprising, as radiotherapy is recommended by guidelines for the treatment of pain associated with metastatic bone disease [16, 17], a symptom experienced by up to 90 % of patients in the later stages of metastatic cancer [16]. A large review of worldwide radiation practice patterns found that multiple fractions of radiation therapy were preferred to single fractions to treat pain associated with bone metastases in Europe [18]. Both multiple-fraction and single-fraction treatment regimens have been shown to be equally effective in palliating pain [19]; therefore, encouraging the use of single-fraction therapy may help to reduce outpatient- and procedure-related HRU.

The HRU associated with SREs reported here, and the subsequent implications for costs [11, 12, 20, 21], highlight the potential reduction in HRU that could be achieved using BTAs to prevent SREs. Indeed, European guidelines recommend BTAs, such as bisphosphonates and denosumab, for patients with bone metastases secondary to advanced malignancies [17, 22–26]. In patients with breast cancer, ibandronate significantly decreased the incidence of new bone events by 38 % and delayed the time to first SRE compared with placebo (50.6 vs. 33.1 weeks, respectively) [27]. Similar delays in the time to first SRE were observed in patients with breast cancer receiving pamidronate compared with those receiving placebo [28]. In patients with prostate cancer, zoledronic acid significantly delayed the time to first SRE, and reduced the ongoing risk of SREs by 36 %, compared with placebo [29]. A recent study reported that ibandronate was inferior to zoledronic acid for reducing the frequency of SREs in patients with breast cancer [30], and another study reported that zoledronic acid significantly decreased the risk of developing an SRE by an additional 20 % compared with pamidronate [31]. In addition, denosumab was shown to be superior to zoledronic acid in delaying or preventing SREs in patients with advanced solid tumors [32–36].

Despite the potential benefits of treatment and contrary to guidelines and published evidence, our study found that only 40 % of patients were receiving bisphosphonates at baseline. After experiencing an SRE, 37 % of patients remained untreated and studies suggest that these patients were at risk of further SREs [29, 37] and subsequent resource use. Our data reflect the findings of a large European patient chart audit in which only 53 % of patients with bone metastases received BTA treatment. Furthermore, the audit indicated that 17 % were expected never to receive treatment, reflecting a possible gap in patient care [38].

The patient populations in this study differed from those in clinical trials. Indeed, in clinical trials of BTAs in patients with bone metastases secondary to cancer, asymptomatic SREs are often captured by regular examinations, including as bone scanning, used during follow-up [32, 33, 39]. Recent clinical trials have used an alternative set of endpoints, referred to as symptomatic skeletal-related events (SSEs), comprising radiation to bone, symptomatic pathologic fracture, surgery to bone and symptomatic spinal cord compression [1, 40]. It is likely that all SSEs were captured in the present study, but that some asymptomatic SREs may not have been identified owing to the absence of regular scans.

The main limitation of this study is the probable underestimation of SRE-associated HRU. In this study, most patients had one or two sites of bone metastasis; therefore, the HRU for individuals with multiple bone metastases was probably not fully captured because these patients may require more HRU than those with fewer sites of metastasis. In addition, HRU outside the hospital setting, such as that associated with home visits, is difficult to assess and was not captured in this study. Home visits have been shown to be particularly important in the management of pathologic fractures [9], and may also become important for other SREs, such as spinal cord compression, owing to the loss of mobility and independence that occurs as metastatic bone disease progresses. Furthermore, patients with more advanced disease may be treated in a hospice facility, and hospice HRU may not have been fully captured in our study.

Another limitation of this study is that the distribution of SREs may not accurately reflect that of a real-world setting, owing to recruitment according to predefined targets (in some instances target numbers of patients were not met). In addition, at the Finnish centre, selection bias was observed for radiation to bone. Patients receiving this treatment were enrolled only from palliative wards, where associated HRU may have been higher owing to the level of care delivered at this type of facility. The other HRU data from Finland are, however, considered representative. Despite the limitations, this study provides valuable information on the HRU associated with SREs in clinical practice.

In conclusion, this study demonstrates that in real-world practice, SREs are associated with substantial increases in HRU across all countries investigated. Previous estimates of the contributions of pathologic fractures to HRU may have been imprecise, owing to grouping of all fractures together in other studies. The availability of more effective and better tolerated BTAs to prevent SREs may help to reduce the burden placed on healthcare resources. Further studies on the effect of delaying SREs on HRU and costs in real-world practice are warranted.
